# Workplace violence and its strong association with depression among Brazilian workers: insights from a national survey

**DOI:** 10.3389/fpsyt.2025.1534511

**Published:** 2025-10-31

**Authors:** Alexandre Faisal-Cury, Alicia Matijasevich, Daniel Maurício de Oliveira Rodrigues, Karen M. Tabb, Maria Fernanda Peres

**Affiliations:** ^1^ Departamento de Medicina Preventiva, Faculdade de Medicina, Universidade de São Paulo, São Paulo, SP, Brazil; ^2^ School of Social Work, University of Illinois at Urbana-Champaign, Urbana, IL, United States

**Keywords:** workplace violence, violence at work, depressionve symptoms, mental health, workers, occupational health, exposures, work-related exposure

## Abstract

**Objective:**

We aimed to investigate the association between workplace violence and depression among Brazilian workers.

**Methods:**

We used a cross-sectional study using data from the Brazilian National Survey (PNS, 2019), a population-based study. A sample of 52,475 workers (18–98 years old; N = 22,797 women and 29,678 men) answered a questionnaire covering sociodemographic data, workplace violence (psychological and physical or sexual) in the last 12 months, and the Patient Health Questionnaire-9. Logistic regression models were performed and adjusted for sociodemographic factors. All analyses were conducted using weighted and stratified data by gender.

**Results:**

The prevalence of depression in this group of workers was 15.8% for women and 5.7% for men. Workplace psychological violence was reported by 4.4% of women and 4.9% of men. For physical or sexual violence, the figures were 0.5% for women and 0.6% for men. All types of violence were significantly associated with depression for women (psychological: OR: 3.37, 95% CI 2.31–4.93 and physical/sexual: OR: 2.44, 95% CI 1.15–5.15) and for men (psychological: OR: 4.07, 95% CI 3.13–5.27 and physical/sexual: OR: 3.88, 95% CI 1.06–5.27). Both types of workplace violence interacted with gender without noticeable differences between men and women.

**Conclusion:**

Workplace violence is associated with depression in both sexes. Preventive and management strategies for workplace violence are recommended.

## Introduction

Depression is one of the leading factors of disability worldwide, with approximately 350 million people living with depression across the globe—a number that steadily increases each year (1). While depression is a major issue globally, the majority of the data are limited to high-income countries. Low- and middle-income countries (LMICs) include countries such as Brazil, and these are rarely described in global mental health estimates (2, 40). (Crisp, 2022). In Brazil, the prevalence of depression varies between 5% and 10% (3), which positions the country on the list of nations with the highest number of individuals affected by depression. Strategies to prevent, reduce, and identify the burden of depression early at the population level are essential and may vary based on country context. For example, in LMICs such as Brazil, universal healthcare access might create improved access to care compared to other LMICs that do not have universal healthcare.

Strategies to prevent and reduce depression might be tailored by gender. Epidemiological research has shown substantial gender-related differences in the prevalence of depression, with 170 per million women versus 109 per million men (4) living with depression globally. Among workers, an estimated 12 billion workdays are lost each year due to depression and anxiety, with a cost of 1 trillion dollars (USD) annually in terms of productivity loss. According to the World Health Organization, approximately 15% of working-age adults experience a mental disorder at some point (5). The causes of depression include complex interactions between social, psychological, and biological factors. Currently, with the acknowledgment of employment as a significant contribution to an individual´s physical and mental well-being (6), there is considerable interest in exploring the role of workplace violence as a risk factor for depression. Previous studies have shown that work-related violence predicts depressive symptoms (7) and antidepressant treatment (8, 9).

Systematic reviews with cross-sectional and longitudinal studies on the association of violence and poor mental health have also reported an elevated risk of depression among persons exposed to workplace violence (WPV) (10, 11). According to the International Labor Office (13), the definition of WPV is “any action, incident, or behavior that departs from reasonable conduct in which a person is assaulted, threatened, harmed, injured in the course of, or as a direct result of, his or her work.” Defining violence as either physical (such as attacks and beating) or psychological (such as threats and harassment) can be problematic, as the two types often overlap and vary in intensity. However, the aforementioned reviews also reported that most of the studies had methodological limitations, bias, and confounding. One particular concern is selection bias due to the potential selection of individuals with higher depression vulnerability into occupations with a high risk of violence (9). Another issue is the use of symptom scales for the measurement of depressive symptoms instead of a diagnostic criterion (10). Additionally, many studies have focused on a specific group of workers, such as health care workers (12), rather than the general population. Finally, the strong heterogeneity and the lack of a clear definition of WPV across studies make comparisons more challenging. In Brazil, a population-based study of workers (19,450 men and 16,992 women) found that WPV was a significant risk factor for major depressive disorder among women and was borderline significant among men (P = 0.08). Given this finding, it is essential to identify associated factors between workplace violence and depression for the purpose of prevention and reducing the burden of unmet mental health needs.

The identification of occupational risk factors that are subject to preventive actions is essential for the physical and mental well-being of workers. WPV is a potential target for intervention and primary prevention of depression. In Brazil, the National Policy on Workers’ Health aims to strengthen surveillance and promote the health of workers, fostering a healthy work environment and identifying health problems and environmental risk factors more effectively. Based on this diagnosis of the need for healthy workplaces, the adoption of interventions in work environments and processes is suggested, aiming to improve the quality of life for workers. Accordingly, the objective of this research was to investigate the association between WPV and depression using data from the 2019 National Health Survey (Pesquisa Nacional de Saúde [PNS]). This study examines workplace violence in the general Brazilian workforce across all occupations, not limited to healthcare settings. Our exposure is violence that occurred at the workplace, whereas violence occurring outside the workplace is treated as a separate covariate and is not considered WPV.

## Methods

### Design and sample

Our study employed a cross-sectional design that encompassed a representative sample of residents in private households across Brazil. We use data from the PNS 2019 (March to August), which is a subset of the Master Sample of the Integrated Household Survey System from the Brazilian Institute of Geography and Statistics. The survey employed a three-stage cluster sampling design: (1) primary sampling unit selection through simple random sampling, (2) selection of permanent private households within each primary sampling unit through simple random sampling, and (3) selection of a resident age 18 or older within each household through simple random sampling. Further details on the survey design and methodology can be found in previous publications (14). The data collection was carried out through interviews conducted by trained interviewers using a questionnaire inserted in a mobile data collection device. The PNS questionnaire is organized into 26 thematic modules covering diverse aspects of health and living conditions (e.g., chronic diseases, lifestyle behaviors, oral health, reproductive health, medication use, and health service utilization). For the present study, we selected variables from the modules on mental health (PHQ-9), violence, and sociodemographic characteristics, as they were directly related to our research question. The remaining modules were not included in the analyses because they addressed health topics outside the scope of this investigation. To ensure precision in estimating prevalence rates with 95% confidence intervals (CI) for the targeted behaviors, the sample size was calculated, considering the clustered sample in multiple stages. Additionally, a 20% non-response rate was considered, aiming to maintain a significant two-sided level of 5% and a statistical power of 80%. We selected 88,531 participants (> 18 years old) who responded to the Selected Resident questionnaire of the PNS, with 52,475 reporting having some occupation and being used for our analyses. The response rate for the PNS 2019 was 91.9%. All people who were included in the final sample agreed to participate in the study and signed the informed consent form.

### Main outcome

The Patient Health Questionnaire-9 (PHQ-9) was used to measure depressive symptoms. The instrument assesses the presence and intensity of each of the nine items in the 2 weeks preceding the interview. Scores range from 0 (“not at all”) to 3 (“nearly every day”), and the total score can vary from 0 to 27. Scores of 10 or more are considered cases of major depression, according to Brazilian validation of the instrument (15). We categorized participants into two groups: no depression (score ≤ 9) and depression (score > 9). This cutoff optimizes the sensitivity and specificity of the PHQ-9 (16). The reliability coefficient, Cronbach’s alpha, for the total PHQ-9 score was 0.84 (17).

Individual items of the PHQ-9, including question #9 on suicidal ideation, were not analyzed separately.

### Main exposures

The exposure variable was workplace violence (WPV). The PNS 2019 module on violence records both the type of aggression and the place of occurrence. The PNS 2019 module “V” addresses questions about violence grouped into categories: physical (push, punch, asphyxiate, etc.), psychological (offend, humiliate, etc.), and sexual (touched or manipulated your body or forced you to have sex or any other sexual acts against your will, etc.). We defined WPV strictly as events whose place of occurrence was the workplace. Violence occurring outside the workplace was not considered WPV and was included as an adjustment covariate (“violence outside of work”: yes/no). For the analyses, we used two WPV categories due to the low prevalence of some subtypes when considered separately: psychological violence *and* physical/sexual violence. Exposure to each WPV category was given the value “yes” if at least one listed act in that category occurred at the workplace in the prior 12 months.

### Sociodemographic variables

The sociodemographic characteristics included in this study were gender (man; woman), self-reported race (white, black, brown, other, and indigenous), age group (18–29, 30–44, 45–59, and 60 and above), education level (no education/incomplete elementary/complete elementary; incomplete high school/complete high school; incomplete college/complete college), per capita income (up to 50% minimum wage; > 50% to 100% minimum wage; >100% to 200% minimum wage; > 2 minimum wage), type of work (domestic worker, military, private sector employee, public sector employee, employer, self-employed, unpaid worker), and self-reported experience of additional violence outside of the work place (psychological or physical/sexual) (yes or no).

### Statistical analysis

A descriptive analysis was conducted, and all variables were categorized. Data were shown for men and women. Logistic regression models were employed to estimate crude and adjusted odds ratios (ORs) along with their respective 95% CIs for the association between the two types of WPV and depression. The adjusted multivariate model for each type of violence included variables that were significantly associated with our outcome in the bivariate analysis (p-values < 0.05). The multivariate analysis was performed using the stepwise backward procedure. Thus, variables that initially had p-values ≤ 0.20 in the bivariate analysis were, simultaneously, included in the model. Variables with p-values > 0.05 were progressively removed from the model. The final model included only variables with p-values < 0.05. The differences between men and women in the association between WPV and depression were analyzed with interaction tests. Gender-interaction terms were analyzed with the total sample of participants (men and women). Statistical analyses were conducted using STATA 16 software. We applied the *svy command*, which specifies survey data analysis procedures in Stata, to incorporate the complex sampling design of the PNS 2019 (i.e., stratification, clustering, and sampling weights) and produce population-level weighted estimates. All estimates were weighted based on the sample complexity of the PNS 2019 (weighted total sample size = 159,171,311).

### Ethical aspects

The PNS received ethical approval from the National Committee for Research Ethics (Conselho Nacional de Ética em Pesquisa) within the National Health Council (Conselho Nacional de Saúde): approval was granted in August 2019 under the reference number 3.529.376. Participation in the survey was entirely voluntary, with respondents having the option to complete the questionnaire either partially or in full. No monetary or material remuneration was provided. All participants gave informed consent prior to the interview. Data from the PNS are accessible on the Brazilian Institute of Geography and Statistics website, ensuring the anonymity of participants by excluding any personally identifiable information.

## Results

Among the 88,531 individuals aged 18 and older who responded to the selected resident module, 52,475 (N = 22,797 women, 29,678 men) reported being employed during the reference period. The average age of the participants was 42.2 years, ranging from 18 to 98 years. For both sexes, the predominant age group was 30–44 years (31.6% for both women and men). The self-reported races “brown” and “white” were the most prevalent for both men and women. The majority of participants had a per capita income > 1 to 2 minimum wage (32.9% for women and 31.7% for men). The most common type of work for both women and men was employment in the private sector (35.3% and 47.7%, respectively). The prevalence of depression in this group of workers was 11.0% (95% CI 10.5–11.5), with 15.8% (95% CI 14.8–16.7) for women and 5.7% (95% CI 5.2–6.1) for men. Descriptive analysis of sociodemographic data is presented in **Table 1**. Psychological and physical/sexual violence at work were reported by 4.4% and 0.5% of women, respectively, and by 4.9% and 0.6% of men, respectively (**Table 1**).

**Table 1:** Characteristics of participants in the Brazilian National Health Survey (Pesquisa Nacional de Saúde - PNS), Brazil, 2019.

Variables	Women		Men		
	N	%	N	%	P Level
Depression					<0.001
No	19.149	84.2	27.983	94.3	
Yes	3.648	15.8	1.695	5.7	
Age (years)					<0.001
18/29	4.181	20.4	5.588	22.9	
30/44	9.322	31.6	11.4611	31.6	
45/59	7.178	25.0	9.065	25.5	
60/Max	2.116	23.0	3.564	20.0	
Schooling					<0.001
Elementary	6.858	33.2	13.149	40.6	
High School	8.677	38.5	10.509	38.4	
College	7.262	28.3	6.020	21.0	
Race					0.023
White	8.570	44.5	10.687	43.3	
Black	2.612	12.2	3.621	11.6	
Brown	11.268	42.0	14.903	43.5	
Other	181	0.9	227	0.9	
Indigenous	164	0.4	235	0.7	
Per capita income (minimum wage)					0.55
> 2 MW	5.579	24.6	7.118	25.2	
> 1 to 2 MW	6.521	32.9	8.522	31.7	
> 1/2 to 1 MW	6.271	27.0	7.699	26.8	
Up to 1/2 MW	4.463	15.5	6.335	16.3	
Type of work					<0.001
Domestic worker	3.736	16.8	442	1.0	
Military	57	0.1	483	1.4	
Private sector employee	7.610	35.3	12.993	47.7	
Public sector employee	4.067	14.9	2.805	8.0	
Employer	560	3.1	1.309	5.4	
Self-employed	6.247	27.7	11.502	35.6	
Unpaid worker	520	2.1	203	0.9	
Violence outside of work					<0.001
No	18.822	83.0	25.981	87.3	
Yes	3.957	17.0	3.697	12.7	
Psychological Violence					0.15
No	21,767	95.6	28.281	95.1	
Yes	1.030	4.4	1.397	4.9	
Physical/Sexual Violence					0.40
No	22.708	99.5	29.503	99.4	
Yes	89	0.5	175	0.6	

As shown in **Table 2**, for women, the sociodemographic variables showing higher prevalence of depression were self-reported race “indigenous” (20.4%), having elementary schooling (18.6%), and per capita income up to 50% of minimum wage (19.5%). For men, the sociodemographic variables showing a higher prevalence of depression were self-reported race “other” (9.8%) and being engaged in unpaid work (7.2%). Depression was more prevalent among women (33.4%) than men (13.7%) who reported violence outside of work. Depression was prevalent among men who reported psychological violence at work (15.1%, 95% CI 12.2–18.4) and physical/sexual violence at work (18.9%, 95% CI 11.1–30.4) and was highly prevalent among women who reported psychological violence at work (30.2%, 95% CI 22.9–38.6) and physical/sexual violence at work (30.4%, 95% CI 17.7–47.0). In the bivariate analysis, the following variables were associated with a higher risk of depression among women: lower per capita income, elementary schooling, violence outside of work, and all types of violence at work (psychological: OR: 2.43, 95% CI 1.65–3.57 and physical/sexual: OR: 2.34, 95% CI 1.15–4.78). For men, the variables associated with a higher risk of depression were type of work, with all types except military showing a higher risk in comparison with domestic work; experiencing violence outside of work; and all types of violence at work (psychological: OR: 3.26, 95% CI 2.53–4.20 and physical/sexual: OR: 3.96, 95% CI 2.10–7.46) (**Table 2**).

**Table 2:** Characteristics of participants and unadjusted odds ratio (OR) with 95% confidence intervals of the association between sociodemographic variables and workplace violence with depression in the Brazilian National Health Survey (Pesquisa Nacional de Saúde - PNS), Brazil, 2019.

Variables	Women			Men		
	Depression (%) (IC 95%)	(OR – IC 95%)	p	Depression (%) (IC 95%)	(OR – IC 95%)	P
Age (years)			0.09			0.40
18/29	17.3 (15.3 – 19.4)	1		5.8 (4.8 - 6.9)	1	
30/44	15.6 (14.3 – 17.0)	0.88 (0.74 – 1.04)		5.7 (5.0 - 6.5)	0.98 (0.77 – 1.24)	
45/59	16.7 (15.3 – 18.2)	0.96 (0.80 – 1.14)		6.0 (5.1 – 6.9)	1.03 (0.80 – 1.32)	
60/Max	13.5 (10.9 – 16.7)	0.74 (0.55 – 1.00)		4.9 (3.7 – 6.4)	0.83 (0.59 – 1.17)	
Schooling			<0.001			0.90
Elementary	18.6 (16.8 – 20.6)	1		5.9 (5.2 – 6.6)	1	
High School	14.4 (13.1 – 15.8)	0.73 (0.62 – 0.87)		5.1 (4.4 - 6.0)	0.87 (0.70 – 1.06)	
College	14.2 (12.8 – 15.8)	0.72 (0.60 – 0.86)		6.0 (5.0 – 7.2)	1.02(0.81 – 1.28)	
Race			0.20			0.81
White	14.8 (13.5 – 16.3)	1		5.5 (4.9 - 6.3)		
Black	16.5 (14.1 - 19.1)	1.13 (0.91 – 1.40)		6.9 (5.6 - 8.4)	1.25 (0.97 – 1.61)	
Brown	16.6 (15.1 – 18.2)	1.14 (0.97 – 1.33)		5.2 (4.6 - 6.0)	0.94 (0.78 – 1.14)	
Other	9.4 (4.7 – 17.9)	0.59 (0.28 – 1.26)		9.8 (1.9- 38.1)	1.84 (0.32 – 10.4)	
Indigenous	20.4 (11.4 – 33.7)	1.47 (0.73 – 2.95)		4.9 (2.3 - 9.8)	0.87 (0.40 – 1.86)	
Per capita income (minimum wage)			<0.001			0.47
> 2 MW	12.1 (10.6 - 13.7)	1		5.5 (4.7 - 6.4)	1	
> 1 to 2 MW	15.2 (13.5 - 17.2)	1.30 (1.06 – 1.60)		5.4 (4.5 - 6.4)	0.98 (0.76 – 1.25)	
> 1/2 to 1 MW	17.5 (15.7 - 19.6)	1.54 (1.27 – 1.87)		5.9 (5.0 - 6.9)	1.08 (0.85 – 1.37)	
Up to 1/2 MW	19.5 (17.5 - 21.7)	1.76 (1.45 – 2.14)		5.8 (4.7 - 7.1)	1.06 (0.81 – 1.38)	
Type of work			0.65			0.054
Domestic worker	17.0 (14.7 – 19.5)	1		2.2 (1.1 - 4.5)	1	
Military	16.7 (5.1 - 42.4)	0.98 (0.26 – 3.64)		3.5 (2.0 - 6.1)	1.57 (0.62 – 3.96)	
Private sector employee	15.7 (14.0 - 17.5)	0.90 (0.72 – 1.13)		5.3 (4.7 - 6.0)	2.43 (1.17 – 5.01)	
Public sector employee	14.1 (12.1 – 16.4)	0.80 (0.63 – 1.02)		6.0 (4.7 - 7.7)	2.78 (1.30 – 5.93)	
Employer	14.5 (10.1 – 20.2)	0.82 (0.53 – 1.28)		5.0 (3.4 – 7.4)	2.28 (1.00 - 5.18)	
Self-employed	16.0 (14.3 – 17.7)	0.93 (0.74 – 1.15)		6.1 (5.3 – 7.1)	2.82 (1.36 – 5.85)	
Unpaid worker	17.4 (12.1 - 24.3)	1.02 (0.65 – 1.62)		7.2 (3.5 – 14.0)	3.33 (1.87 – 9.39)	
Violence outside of work			<0.001			<0.001
No	12.1 (11.2 – 13.1)	1		4.4 (4.0 – 4.9)	1	
Yes	33.4 (30.3 – 36.5)	3.62 (3.04 – 4.31)		13.7 (11.8 – 16.0)	3.41 (2.77 -4.19)	
Psychological Violence			<0.001			<0.001
No	15.1 (14.2 – 16.0)	1		5.1 (4.7 - 5.6)	1	
Yes	30.2 (22.9 – 38.6)	2.43 (1.65 – 3.57)		15.1 (12.2-18.4)	3.26 (2.53 -4.20)	
Physical/Sexual Violence			<0.001			<0.001
No	15.7 (14.8 – 16.6)	1		5.5 (5.1 - 6.0)	1	
Yes	30.4 (17.7 – 47.0)	2.34 (1.15 – 4.78)		18.9 (11.1 - 30.4)	3.96 (2.10 – 7.46)	

In the final adjusted model, all types of violence were significantly associated with depression for women, after adjusting for schooling and violence outside of work (psychological: OR: 3.37, 95% CI 2.31–4.93 and physical/sexual: OR: 2.44, 95% CI 1.15–5.15). Similarly, for men, after adjusting for type of work and violence outside of work, all types of violence were significantly associated with depression (psychological: OR: 4.07, 95% CI 3.13–5.27 and physical/sexual: OR: 3.88, 95% CI 1.06–5.27) (**Table 3**).

**Table 3:** Adjusted odds ratio (OR) with 95% confidence intervals of the association between workplace violence and depression in the Brazilian National Health Survey (Pesquisa Nacional de Saúde - PNS), Brazil, 2019.

Variables	Depression			
	Women		Men	
	OR Adjusted (IC95%)	P	OR Adjusted (IC95%)	P
Psychological Violence *; **		<0.001		<0.001
No	1		1	
Yes	3.37 (2.31 – 4.93)		4.07 (3.13 – 5.27)	
Physical/Sexual Violence *; **		0.019		<0.001
No	1		1	
Yes	2.44 (1.15 – 5.15)		3.88 (1.96 – 7.65)	

Women: * adjusted for scholling and violence outside of work.

Men: ** adjusted for type of work and violence outside of work.

The study of interaction tests showed that the association of violence at work with depression did not differ between genders. The interaction term between psychological (OR interaction term = 0.81, 95% CI 0.51–1.29, P = 0.38) and physical/sexual (OR interaction term = 0.63, 95% CI 0.23–1.74) violence and sex in the fully adjusted logistic regression model were not significant, indicating that sex did not moderate the association between WPV exposure and the likelihood of developing depression.

## Discussion

The results of the present study, based on population-based survey data, showed that the prevalence of depression among Brazilian workers is 15.8% for women and 5.7% for men. In the workplace, psychological violence was the most common type of violence (4.4% for women and 4.9% for men) while physical/sexual violence was much less prevalent (0.5% for women and 0.6% for men). All types of violence at work were significantly associated with depression among workers.

Workplace violence (WPV) is a relevant public health issue in many countries, including high- and middle-income settings such as the Republic of Korea (18) Denmark (19, 20), Italy (21), and Brazil (22). Comparing the prevalence of WPV between studies is difficult as they adopt different definitions and terminologies regarding the occurrence of violence related to the workplace. The current and growing interest in the topic is justified by the various negative consequences of violence on functioning at work, such as malpractice, low quality of care, absenteeism, high turnover (21), and workers’ health. Previous meta-analyses have explored the associations between WPV and depression. Overall, they showed that violence and threats thereof at the workplace are associated with increased risk of mental ill health (22).

Recently, Rudkjoebing et al. included 14 cross-sectional and 10 cohort studies to summarize the risk of depression among workers exposed to violence. Depressive and anxiety symptoms, burnout, and psychological distress were examined in 17 studies. The researchers found an elevated risk of depression among the exposed (RR 1.42, 95% CI 1.31–1.54] (20).

In Brazil, da Silva et al. (12) evaluated 3,141 primary health care workers in the city of São Paulo, reporting an association between WPV and depressive symptoms, as well as probable major depression. Of note, they assessed different types of exposure, including insults, threats, physical aggression, and witnessing violence, which were strongly and progressively associated with depressive symptoms (the adjusted OR was 1.67 for exposure to one type of violence and 5.10 for exposure to all four types). Another Brazilian study by Oenning et al. used data from the PNS 2013 and explored the associations between a large set of occupational factors, including violence and depression in the Brazilian working population. They reported a significant risk of depression for women submitted to WPV. WPV was assessed with only two items related to location and perpetrator (6). Differently from Oenning et al.’s study, we used the latest version of the PNS (2019), which provides a much broader and more detailed assessment of violence. Overall, comparisons between studies are difficult since there is great variation in the prevalence of WPV according to occupational setting, definitions, and measurement methods.

Few causal mechanisms have been cited to explain the development of depression among individuals undergoing WPV. In addition to the feelings of helplessness and hopelessness that are common in this type of situation, experiencing violence can affect certain characteristics and behaviors of the person, such as coping strategies, sense of coherence, and sense of efficacy. These effects may, in turn, increase the risk of workers experiencing anxiety and depression (23). This perspective is consistent with theoretical frameworks that conceptualize WPV as a psychosocial stressor that undermines individual resources and increases vulnerability to mental health problems (23, 41, 42).

There is evidence that stress resulting from the experience of violence has the potential to deregulate the autonomic nervous system, leading to emotional disorders (24, 25). Nevertheless, the development of depression after violence is also influenced by other factors, such as personality, coping strategies, and social abilities (26).

In addition to these theoretical perspectives, empirical evidence from different occupational settings further illustrates the wide-ranging consequences of WPV. In the retail sector, Konda et al. (43) demonstrated that approximately 28% of non-robbery-related occupational homicides occurred as a result of conflicts, with security workers in bars being one of the groups at highest risk. In education, Tiesman et al. (44) highlighted that physical assaults against education workers negatively affect their quality of life, job satisfaction, retention, and professional performance, while López-Vílchez et al. (45), in a study conducted in Spain, linked workplace bullying in schools to the deterioration of teachers’ mental health. In the service sector, Baek et al. (46) showed that the implementation of institutional protection systems, such as regular counseling, grievance committees, and stress-relief programs, significantly reduced the occurrence of workplace violence in the Republic of Korea. When present, violence was associated with severe psychological consequences, including depression, insomnia, anxiety, exhaustion, and post-traumatic stress disorder. Furthermore, Duan et al. (47) evidenced that workplace violence is directly associated with reduced job satisfaction, burnout, and turnover intention, with social support playing an important mediating role in mitigating these adverse effects. Collectively, these findings demonstrate that the deleterious effects of workplace violence—physical, psychological, and organizational—extend across multiple professional sectors, strengthening the discussion in our manuscript and aligning it with the broader scope of the WPV phenomenon.

Our results show that the effect of WPV on depression does not vary by gender. Our results indicate that women exposed to WPV are more likely to report depressive symptoms than men. Psychological and sexual WPV were also strongly associated with depression, particularly among women. In addition, differences by schooling and race suggest that social inequalities further shape the mental health consequences of WPV. These findings highlight the importance of gender- and equity-sensitive approaches in preventing and addressing WPV. Previous studies about the influence of gender in the relationship between bullying and mental health found mixed evidence about the moderating effect of gender in the association between violence and depression. A few studies have reported that a negative impact on mental health was more evident among men (27, 28), while others have found that being a man was a protective factor (29, 30). The better mental health of men may reside in the way they cope with stressors (30). Sex-related differences in the effects of violence on mental health may be explained by differences in brain function. Previous studies have already shown sex-related differences in the brain function that underlies emotional expression and regulation (31, 32). Recently, Dark et al. found that reactivity of specific brain areas and circuits (amygdala, parahippocampal gyrus, dorsolateral prefrontal cortex, and inferior parietal lobule) to threat or violence exposure occurs among women, but not men. They suggested that gender differences in threat-related brain and psychophysiological activity may have implications for mental health (33). Although sociodemographic characteristics such as schooling and race were associated with depression in our results, the main objective of this study was to examine the relationship between workplace violence and depression. Therefore, these additional correlates were not explored in depth, as our discussion prioritized the central exposure–outcome association.

The present study has some strengths, notably the use of information derived from a population-based survey with a large number of participants, included through a complex probabilistic sampling process. Unlike previous versions, information about violence in the PNS 2019 includes a variety of questions and aspects that provide a comprehensive view of the problem in the country.

Despite these strengths, this study carries certain limitations. Firstly, as it is a cross-sectional study, causality cannot be inferred. This design does not allow us to establish temporality between WPV and the development of depression among workers, and reverse causation cannot be ruled out. In other words, we can not evaluate whether WPV caused depression among the workers, or if depression preceded the violence. Secondly, misclassification of the exposure related to the outcome may have occurred, as depressed individuals might be more inclined to report workplace violence. This bias could lead to an overestimation of the association between violence and depression. Thirdly, depression was assessed through self-report using a questionnaire (PHQ-9). It is acknowledged that measurement of clinical depression in epidemiological studies is challenging, and the use of a psychiatric interview would be the more accurate method for a psychiatric assessment. Nevertheless, its implementation in population surveys with a large number of participants is logistically challenging and costly. Additionally, we used the PHQ-9, a validated diagnostic instrument based on the fourth edition of the Diagnostic and Statistical Manual of Mental Disorders criteria, to measure depression. Fourthly, selection bias may also play a role, particularly the healthy worker effect. Workers who experienced violence may have left their jobs, while those not exposed to violence remained in the workforce, potentially leading to an underestimation of the true association. In addition, we focused only on sociodemographic covariates, so residual confounding by other unmeasured factors cannot be excluded. Furthermore, some methodological challenges previously discussed in the Introduction could not be fully addressed in our study, including heterogeneity in definitions and measurement of WPV across countries, which limits international comparability of findings. Finally, regarding the generalizability of our results, we excluded from our analysis workers under 18 years of age, and we lacked information about informal workers. Both groups constitute a significant portion of the Brazilian workforce and are more likely to face poor working conditions and lower wages, which may increase the risk of both violence and depression.

Our findings underscore the need to first identify and then develop strategies to prevent WPV, as this may be associated with depression and other health problems. Notifications of violence, which are compulsory in Brazil, are essential for recognizing the severity of the problem and supporting public policies focused on the prevention of violence and promotion of worker health (34, 35). When planning actions based on the recognition of WPV, guidance is provided in the National Occupational Health Policy (36). Preventive interventions include staff training in the management of WPV and implementation of a systematic approach to risk management (37, 38). Appropriate support provided for victims of WPV is also recommended. A scoping review of 11 studies found that interventions carried out by psychiatric nurses and psychologists (training programs, cognitive behavior therapy, and workplace violence programs) reduce anxiety and depression in victims of WPV (39).

## Conclusion

Our findings showed that WPV is associated with depression in both genders. Depression represents a prevalent public health concern with many consequences for workers, yet it is also responsive to effective and safe treatment. Comprehensive health care for workers with depression should include assessment of WPV. Addressing WPV adequately necessitates timely recognition and the development and implementation of preventive and management strategies for threats and aggression. Moreover, it entails providing organizational and psychological support for victims. By taking such measures, workplaces can foster healthier and safer environments, ultimately promoting the well-being of their employees.

## Data Availability

Publicly available datasets were analyzed in this study. This data can be found here: https://www.ibge.gov.br/en/statistics/social/justice-and-security/16840-national-survey-of-health.html?edicao=19375.

## Author’s note

This article utilized the Brazilian National Health Survey (Pesquisa Nacional de Saúde - PNS), Brazil, 2019 (https://www.pns.icict.fiocruz.br/questionarios/). The authors have adapted the language for some of the sociodemographic characteristics to be socially correct.
